# *In Cis* Effect of *DMPK* Expanded Alleles in Myotonic Dystrophy Type 1 Patients Carrying Variant Repeats at 5′ and 3′ Ends of the CTG Array

**DOI:** 10.3390/ijms241210129

**Published:** 2023-06-14

**Authors:** Virginia Veronica Visconti, Elisa Macrì, Maria Rosaria D’Apice, Federica Centofanti, Roberto Massa, Giuseppe Novelli, Annalisa Botta

**Affiliations:** 1 Department of Biomedicine and Prevention, Genetics Unit, University of Rome “Tor Vergata”, Via Montpellier 1, 00133 Rome, Italy; virginia.veronica.visconti@uniroma2.it (V.V.V.); elisa.macri314@gmail.com (E.M.); federica.centofanti@gmail.com (F.C.); novelli@med.uniroma2.it (G.N.); 2 Laboratory of Medical Genetics, Tor Vergata Hospital, Viale Oxford 81, 00133 Rome, Italy; d.apice@med.uniroma2.it; 3 Department of Systems Medicine, University of Rome “Tor Vergata”, Via Montpellier 1, 00133 Rome, Italy; massa@uniroma2.it; 4 Istituto di Ricovero e Cura a Carattere Scientifico (IRCCS) Neuromed, Via Atinense 18, 86077 Pozzilli, Italy; 5 Department of Pharmacology, School of Medicine, University of Nevada, Reno, NV 89557, USA

**Keywords:** Myotonic dystrophy type 1 (DM1), *DMPK* gene, variant repeats (VRs), methylation, inheritance

## Abstract

Myotonic dystrophy type 1 (DM1) is an autosomal dominant multisystemic disease caused by a CTG repeat expansion in the 3′-untranslated region (UTR) of *DMPK* gene. DM1 alleles containing non-CTG variant repeats (VRs) have been described, with uncertain molecular and clinical consequences. The expanded trinucleotide array is flanked by two CpG islands, and the presence of VRs could confer an additional level of epigenetic variability. This study aims to investigate the association between VR-containing *DMPK* alleles, parental inheritance and methylation pattern of the DM1 locus. The DM1 mutation has been characterized in 20 patients using a combination of SR-PCR, TP-PCR, modified TP-PCR and LR-PCR. Non-CTG motifs have been confirmed by Sanger sequencing. The methylation pattern of the DM1 locus was determined by bisulfite pyrosequencing. We characterized 7 patients with VRs within the CTG tract at 5′ end and 13 patients carrying non-CTG sequences at 3′ end of the DM1 expansion. *DMPK* alleles with VRs at 5’ end or 3’ end were invariably unmethylated upstream of the CTG expansion. Interestingly, DM1 patients with VRs at the 3′ end showed higher methylation levels in the downstream island of the CTG repeat tract, preferentially when the disease allele was maternally inherited. Our results suggest a potential correlation between VRs, parental origin of the mutation and methylation pattern of the *DMPK* expanded alleles. A differential CpG methylation status could play a role in the phenotypic variability of DM1 patients, representing a potentially useful diagnostic tool.

## 1. Introduction

**Figure 1:**
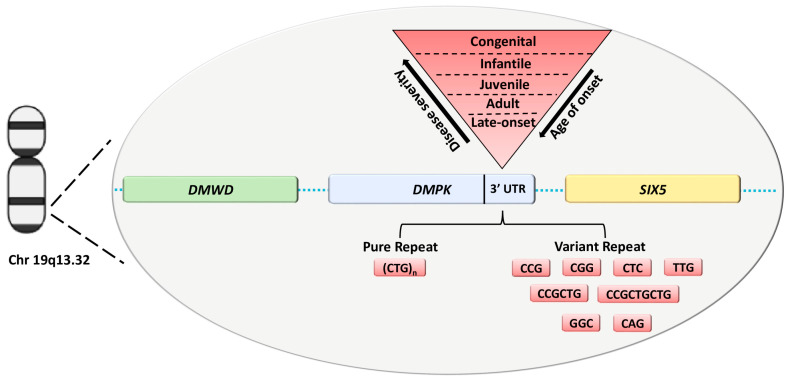
*DMPK* with *DMWD* and *SIX5* neighbor genes located on Chr 19q13.32. The disease-causing mutation is an expansion of a CTG repeat tract in the 3′ UTR of the *DMPK* gene with a variability of repeat units correlated to DM1 clinical phenotypes. DM1 expanded alleles can be structured with pure and variant repeats of the CTG array. Abbreviation: UTR, untranslated region.

Myotonic Dystrophy type 1 (DM1, OMIM #160900) is the most common muscular dystrophy in adults with an estimated incidence of 1:8000 [1]. It is an autosomal dominant disorder characterized by multisystemic clinical features, including weakness and loss of muscle mass, myotonia, cataracts and cardiac conduction defects [2]. The disease-causing mutation is an expansion of a CTG repeat tract in the 3′-untranslated region (UTR) of the DM1 protein kinase (*DMPK*) gene on chromosome 19q13.3 (Figure 1). 

The CTG array is unstable and prone to expand during meiotic transmission, providing a molecular explanation for the anticipation phenomenon defined by increased severity and earlier age of onset over generations [3,4]. At molecular level, the expanded DNA is transcribed in CUG-containing RNA that is able to sequester key splice factors, such as MBNL proteins, leading to the abnormal splicing of several genes that contribute to the variability of DM1 phenotypes [2,5]. To date, a partial overlap between the different clinical DM1 subtypes (late-onset, adult, juvenile, infantile and congenital) and the repeat size ranges was observed (Figure 1) [6,7]. However, the inherited CTG expansion show both a positive correlation with disease severity and an inverse correlation with age of onset [8]. Overall, affected individuals inherit from ∼50 to several thousand CTG repeats [8]. Individuals with CTG repeat numbers between 38 and 49 are considered to carry “premutated” alleles and may remain asymptomatic throughout their lives, constituting the pool from which new premutations and complete DM1 mutations are most likely to arise [9]. However, the exact molecular mechanism leading to phenotypic variability, including age of onset, severity and clinical manifestations of the disease, is still far from being completely understood. Although the size of the expansion seems to play a key role in determining the severity of DM1 phenotype, it is important to emphasize that the somatic mosaicism of the *DMPK* expansion imposes limitations and difficulties in genotype-phenotype correlations. These issues are being overcome by different molecular methods such as small poolpolymerase chain reaction (SP-PCR) or the latest PacBio long-read sequencing technology, enabling to determine progenitor allele estimation (ePAL) and the levels of somatic mosaicism in DM1 patients [10,11,12].

Literature data identify a potential correlation between variability in clinical phenotype and age of onset, and sequence interruptions in the CTG repeat. DM1 alleles containing non-CTG variant repeats (VRs), such as GGC, CTC and CCG, at the 3′ and 5′ end of the CTG have been described in about 3–11% of DM1 patients, percentage likely to increase with the use of third next-generation sequencing methods (Figure 1) [12,13,14,15].

It is now well established that VRs have a key role in reducing disease severity since they are significantly associated with a later age at onset of symptoms [10,16]. Non-CTG sequence interruptions also exert a stabilizing effect of expansion on germline transmission even if the expansion is maternally inherited [10,17,18]. However, downstream molecular effects still need to be completely explored, and the phenotypic variability associated with VRs is huge considering also the significant heterogeneity in terms of pattern, length and location of the interruption along the CTG expansion. These VRs would appear to confer an additional degree of genetic and epigenetic variability. Among epigenetic mechanisms, DNA methylation plays an important role in DM1 pathogenesis, as the CTG repeat tract is embedded in a 3.5 kb CpG island and flanked by two binding sites for the insulator protein CTCF, named CTCF1 and CTCF2 [19,20]. The binding of CTCF is methylation sensitive and it is supposed to establish an insulator element together with the CTG repeats and SIX Homeobox 5 *(SIX5)* enhancer region [21]. Several studies have investigated the relationship between methylation level of the DM1 locus and the presence of these VRs alleles, showing variable *in cis* effects of non-CTG interruptions [20,22,23,24]. The importance of defining a methylation signature is highlighted by the demonstrated stability of DNA methylation levels upstream and downstream of CTG repeats over time [22], indicating the potential use of this epigenetic mechanism as a disease biomarker. 

Our study aims to further investigate the association between the presence of variant expanded *DMPK* alleles at both sides of the CTG array and the methylation pattern of the DM1 locus in a cohort of 20 DM1 patients with VRs. The methylation level of the two CpG islands flanking the DM1 expansion was analyzed and correlated with the structure, the length and the parental origin of *DMPK* variant expanded alleles.

## 2. Results

### 2.1. Description of Study Participants

In this study, we analyzed 20 DM1 patients (Pt 1–20) with suspected VRs (8 males and 12 females), with a mean age of 53.3 ± 17.8 [mean ± standard deviation (SD) with a range of 23–79 years] and a juvenile/adult onset of the disease (Table 1). *DMPK* expanded alleles length ranges from 80 to 990 CTG repetitions. The expanded CTG repeat was on the paternal inherited allele in 10 patients (50%), and in 7 patients (35%) it was on the maternal allele, while it was unknown for 3 patients (15%). We also analyzed 20 DM1 patients (Pt 21–40) with “pure” CTG repeat pattern, including 3 cases with congenital DM (CDM), as controls for methylation analysis. *DMPK* expanded allele ranges from 48 to 1600 CTG repetition. The expanded pure CTG repeat was on the paternal inherited in 5 patients, while it was on the maternal allele in 7 patients. For 8 patients it is not possible establish the inherited of the expanded CTG repeat.

### 2.2. Identification of DM1 Alleles Containing Variant Non-CTG Repeats

**Figure 2:**
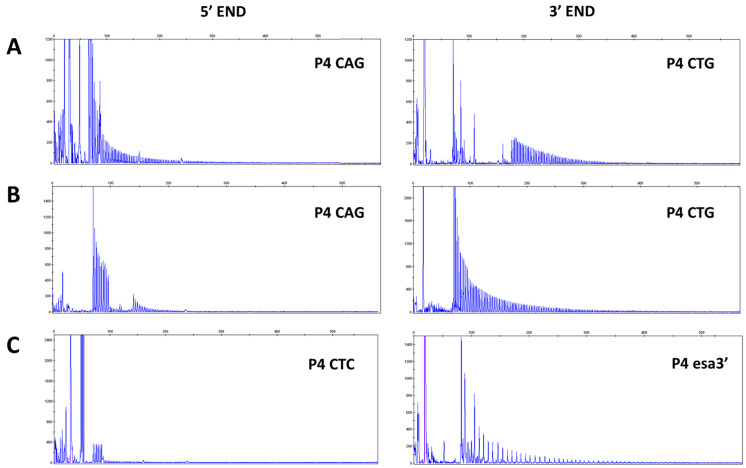
Representative electropherograms showing TP-PCR profiles of DM1 patients with interruptions at 3′ end of CTG array ((**A**), Pt 18) and TP-PCR profiles of DM1 patients which show interruptions at 5′ end of CTG repeats and an expanded 3′ end TP-PCR profile ((**B**), Pt 7). VRs are suspected in the absence of PCR products where (CTG)_5_ and (CAG)_5_ repeated primers cannot anneal. Modified P4 primers allow a first characterization of interruption motifs ((**C**), Pt 3, Pt 17). Abbreviations: TP-PCR, Triplet repeat Primed-PCR; VR, Variant Repeat.

Screening by bidirectional TP-PCR suggested the presence of VRs at 3′ end of CTG expansion in 13 (65%) DM1 patients and at 5′ end in 7 (35%) DM1 patients. In particular, the presence of interruptions is revealed by gaps in the TP profile or drops in signal intensity which might even led to a false negative result if TP-PCR is not performed at both sides of the CTG array. When the 3′ electropherogram profile is interrupted and the TP-PCR at the 5′ end yielded the expected contiguous peak profile, a non-CTG interruption upstream of the CTG stretch is present (Figure 2A). Conversely, patients showing an opposite 3′-5′ TP-PCR pattern carry variant repeats within the CTG expansions at the 5′ end (Figure 2B). To identify the type of variant repeats, samples were further analyzed with additional rounds of TP-PCR, in which the primer P4 CTG/CAG was replaced with one of the primers allowing detection of non-CTG variant repeat motifs described in the literature. TP-PCR reaction performed with P4 esa_3′ and P4 esa_5′ revealed the presence of CCGCTG interruptions within the 3′ end of the expanded alleles in 4 patients and never detected at the 5′ end. TP-PCR performed using primers P4 (CTC) and P4 (CGG)5 also allowed us to identify CTC and CCG motif at the 5′ end in 2 and 1 patients, respectively (examples are given in Figure 2C).

### 2.3. Characterization of DM1 Alleles Containing Variant Non-CTG Repeats 

**Figure 3:**
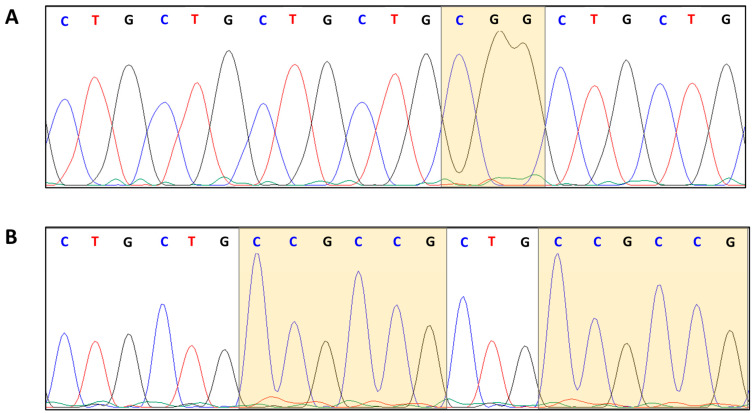
Chromatograms of Sanger sequencing of PCR products confirming the presence of the CGG and (CCG)_n_ VRs: (**A**) CGG at 5′ end (Pt 5), and (**B**) (CCG)_n_ at 3′ end (Pt 18), respectively.

In order to further characterize the CTG array interruption motifs, we used Sanger sequencing to analyse a total of 20 expanded *DMPK* variant alleles, in order to confirm the presence of the interruptions previously identified by TP-PCR and to define their nucleotide compositions (Table 2 and Figure 3). 

**Figure 4:**
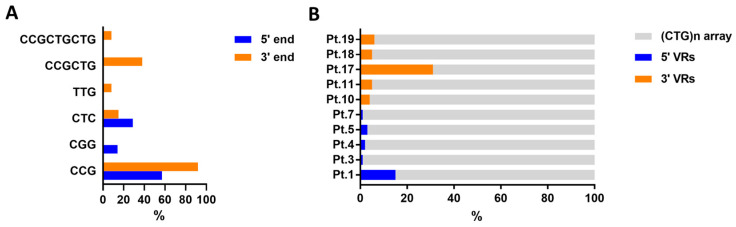
Compositions and lengths of VRs in 5′ and 3′ ends interruption of the CTG array. (**A**) Percentages of different VRs (CCG, CGG, CTC, TTG, CCGCTG, and CCGCTGCTG) identified by Sanger sequencing in all DM1 patients carrying variant *DMPK* alleles at 5′ end (blue) and 3′ end (orange) of the CTG array. (**B**) Percentages of VRs at 5′ (blue) and 3′ (orange) ends in the CTG array in DM1 patients with complete sequence of the expanded allele amplifiable by LR-PCR. Abbreviation: VR, Variant Repeat.

In the current study, 10/20 DM1 patients were characterized over the entire length of the expanded allele amplifiable by LR-PCR. The remaining 10/20 DM1 expanded alleles were only partially characterized by Sanger sequencing of the corresponding TP-PCR products. The following motifs have been identified: CCG, CCGCTG, CTC, TTG, CGG and CCGCTGCTG. Representative Sanger profiles highlighting VRs composed of CGG and CCG, at the 5′ and 3′ end of the CTG array, are shown in Figure 4.

We observed greater variability in the composition of VRs at the 3’ end, than those at the 5’ end of the CTG expanded tract (Figure 4A). The CCG motif was the most recurrent in either 5′ or 3′ interrupted alleles, with a frequency of 57.1% and 92.3%, respectively. Overall, patients with 3′ end interruption showed a higher percentage of variant sequence, reaching up to 31.6% of the expanded repeat tract (Figure 4B).

### 2.4. CpG Methylation Analysis of DMPK Gene by Pyrosequencing Analysis

To assess whether the presence of the previously characterized VRs could modulate *DMPK* methylation pattern, CpG methylation analysis was performed in our cohort of DM1 patients with VRs (Table 3) and in a cohort of DM1 patients carrying “pure” CTG expansions (Table S1).

**Figure 5:**
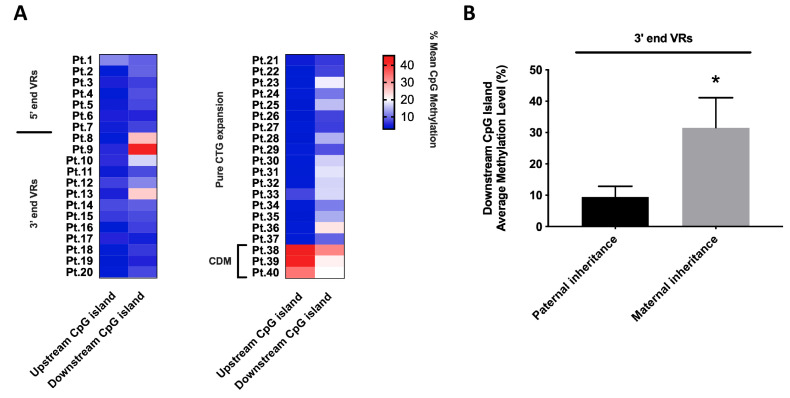
Distribution of average methylation levels (%) of *DMPK* locus among 5′ end VRs, 3′ end VRs and “pure” DM1 patients. (**A**) Heatmaps depicting the average methylation levels in the upstream and downstream islands of the (CTG)n array in VRs and pure DM1 patients. (**B**) Difference in average downstream CpG island methylation levels in DM1 alleles with VRs at the 3’ end, both in paternal and maternal inheritance. Statistical differences were analyzed by non-parametric Mann–Whitney *U*-test (*p* = 0.012; indicated by * for *p* < 0.05).

Methylation analysis was performed on two CpG islands located upstream and downstream of the (CTG)_n_ tract in the *DMPK* 3′-UTR region. The upstream one comprising 10 CpG sites and the downstream one comprising 6 CpG sites. Both regions were analyzed in DM1 patients with VRs alleles (5′ end, *n* = 7 vs. 3′ end, *n* = 13) and with ‘pure’ CTG alleles as the reference group (*n* = 20). DM1 patients carrying VRs at the 5′ and 3′ ends of the (CTG)_n_ array invariably appear unmethylated in the upstream CpG island, as do “pure” non-congenital DM1 patients showing an average methylation level in the range of 3.8–5.6% in both groups (Figure 5A and Table S1). As expected, the three CDM patients carrying pure DM1 expansion are hypermethylated in both upstream and downstream CpG islands (40.4% vs. 24.6%, respectively). In the downstream CpG island, DM1 patients carrying VRs at 5′ and 3′ ends, along with “pure” DM1 patients globally showed an average methylation level in the range of 7.9–13.9%. However, we identified significantly increased downstream methylation levels (range 25.2–42.5%) in three DM1 patients with 3′ VRs (Figure 5A and Table S1). Interestingly, hypermethylation at 3′ end was associated with maternally inherited interrupted *DMPK* alleles. We observed a statistically significant difference between average methylation level of paternal-inherited 3′ VRs alleles, compared to DM1 patients with disease allele mother-inherited (10.5% vs. 31.4%; *p* = 0.012) (Figure 5B). No significant association was found between the average methylation levels of the *DMPK* locus and the CTG expanded allele length in our cohort.

## 3. Discussion

The percentage of VR-containing mutated *DMPK* alleles is actually underestimated since they could not be easily detectable by routine molecular diagnostic approaches. Due to these limitations, the genetic and epigenetic variabilities of *DMPK* expanded locus containing VRs, and its relationship to the variability of the DM1 clinical phenotype, still appear full of gaps. Probably, the complex relationship linking these factors is due to the great variability in the structure of the *DMPK* alleles, including the pattern, length and location of the interruption along the CTG expansion.

In this study, we identified the presence of VRs in 20 DM1 patients, including 13 patients with 3′ end VRs and 7 patients with 5′ end VRs, which has expanded the cohort of our previous study [25] and the current study represents the widest molecular characterization of patients with VRs described to date. Characterization by direct sequencing allowed us to establish the whole or partial compositions of the expanded trinucleotide array amplifiable by PCR-based techniques, identifying six different VRs, i.e., CCG, CCGCTG, CTC, TTG, CGG and CCGCTGCTG. Interestingly, we observed greater variability in the compositions of VRs at the 3′ end, than those at the 5′ end of the CTG expanded tract in which we identified five and three different non-CTG motifs, respectively. In agreement with previous data, we have identified the most abundant presence of CCG motif, either at 5′ or at 3′ ends, configured as individual repeat, CCG block or part of complex motifs such as CCGCTG or CCGCTGCTG [18,26]. In our cohort, the CCG repetition alone is more often represented compared to other studies [12]. Moreover, in fully sequenced *DMPK* expanded alleles, we observed a variety of repeat motif combinations more diverse than what was previously reported in a Northern European population [12].

In order to assess whether the presence of VRs could modulate *DMPK* epigenetic signature, we investigated CpG methylation profile of the regions upstream and downstream of the CTG expanded tract in our cohort of 20 DM1 patients with VRs and 20 DM1 patients with “pure” CTG alleles as a reference group. DM1 patients with VRs at 5’ and 3’ ends were invariably unmethylated upstream of the CTG expansion. However, 3/10 DM1 patients with VRs at the 3′ end showed higher methylation levels in the downstream island of the CTG repeat tract. Consistent with our data, Santoro et al. also observed a polarized pattern of hypermethylation of the region downstream the CTG array in patients with 3’ end interruptions, compared with uninterrupted alleles [20]. *DMPK* hypermethylation in VR-containing alleles was further confirmed in two other studies, although there were no data on the compositions and sizes of the CTG expanded array [22,23]. Unfortunately, in our three DM1 patients showing higher levels of methylation, it was not possible to characterize the entire expanded array, but only a partial sequence obtained after TP-PCR amplification. This limitation does not allow us to understand if there is an *in cis* effect mediated by specific sequences of interruption, analogously to what has been demonstrated in Fragile X syndrome where hypermethylation is triggered by the presence of CGGs [27]. Furthermore, concordant with literature data, we do not find a correlation between the altered methylation pattern in these three subjects and the overall size of the *DMPK* expanded alleles comprised between 400 and 1000 CTGs. Our result is even more interesting when considering the absence of CDM cases in our cohort of DM1 patients with VRs, who seem to be the only ones with an established stable higher methylation signature among “pure” DM1 patients. On the other hand, CDM patients carrying VRs are unlikely to find since interruptions within the CTG array seem to exert a stabilizing effect either in the germinal and in the somatic tissues. We also observed a downstream *DMPK* methylation signature preferentially associated with maternal inheritance, but this finding is limited by the low number of DM1 patients carrying maternally transmitted disease allele with 3′ end VRs. Interestingly, a significant association was also recently identified between maternal inheritance of the disease and higher methylation levels of the downstream island of the CTG array in patients with non-congenital DM1 [22]. In this study seven patients with 3’ end VRs were also included, but their inheritance patterns were not explicitly detailed [22]. All these data point out the difficulty in defining *DMPK* methylation signature, mainly due to the low frequency of DM1 patients with VRs. To date, the mechanism underlying this epigenetic signature in variant *DMPK* alleles is still unclear. One possible explanation could be the presence of a maternal oocyte-specific epigenetic marks leading to aberrant methylation pattern of VRs-containing expanded alleles inherited exclusively from DM1 affected mothers [28].

Our work presents substantial limitations due to the failure to characterize the complete *DMPK* expanded alleles using an amplification-based approach, which has important limitations such as the preference for smaller repeats, the difficulties in the amplification of the GC-rich region and the difficulty of estimating the somatic variation of the repeats. However, these limitations are set to be overcome by PCR-free next-generation sequencing, which has led to discovery of unusual motifs of interruptions within *DMPK* expansions, and also to characterization of DM2 CCTG expansions in *CNBP* up to 50 kb in length [12,13,14,15,29]. This technique would also allow us to define the global methylation pattern of the *DMPK* locus, as previously reported for other repeat expansion diseases such as neuronal intranuclear inclusion disease [30]. A recent study relying on a Clustered Regularly Interspaced Short Palindromic Repeat (CRISPR)/CRISPR-associated protein 9 (Cas9) long-read sequencing allowed the characterization of CTG expansions greater than 1000 CTGs in DM1 patients, thus improving measurement accuracy [14]. Unexpectedly, two patients belonging to the same DM1 family showed expansions with 224 and 360 CNG repeats composed of >85% CCG repeats in association with mild or no muscle symptoms, no cardiac and respiratory impairments [14]. These results are in accordance with literature data correlating VRs with decreased DM1 symptoms [10,31,32].

Although based on routine PCR-based approaches, the current study has expanded the knowledge of genetic and epigenetic variability of the *DMPK* locus in DM1 patients carrying different motifs of non-CTG interruptions. Our results suggest a potential correlation between VRs, parental origin of the mutation and methylation pattern of the *DMPK* expanded alleles that needs further investigations by using the novel long-read PCR-free sequencing technologies. A differential CpG methylation status could indeed play a role in the phenotypic variability of DM1 patients with VRs, representing a potentially useful diagnostic tool that deserves future studies.

## 4. Materials and Methods

### 4.1. Patients Recruitment

The current study has enrolled a group of 20 DM1 patients from Italian families referred to the Medical Genetic Unit of “Policlinico Tor Vergata” for DM1 genetic testing during the period from 2007 to 2021. Informed consent was obtained from all study participants (Ethical Approval register numbers: 232/19 and 61/23) and all experimental procedures were carried out according to The Code of Ethics of the World Medical Association (Declaration of Helsinki). Clinical assessment of all patients, including neurological, ophthalmological, cardiac and laboratory examinations were performed. *DMPK* repeat analysis using a combination of short-range PCR (SR-PCR), bidirectional triplet repeated primed PCR (TP-PCR) and Southern blotting analysis of long range PCR (LR-PCR) following standard diagnostic workflow allowed us to interrogate the presence of an interrupted CTG array [25,32,33]. Twenty DM1 patients carrying “pure” *DMPK* expanded alleles, including three CDM patients, have also included in this study as controls for the analysis of the methylation levels of the DM1 locus.

### 4.2. Bidirectional Triplet Repeated Primed PCR (TP-PCR)

The interruptions of the CTG array were detected with bidirectional TP- PCR (3′ TP-PCR and 5′ TP-PCR) followed by capillary electrophoresis. For interruptions at 5′ end of expansion, internal P4 CAG primer was combined with P2 forward-FAM and P3 primers. For the identification of interruptions at 3′ end P4 CTG primer was combined with P1 reverse-FAM and P3 primer. To identify the type of variant repeats, samples were analyzed with additional TP-PCR, in which the P4 internal primer was replaced with one of the primers allowing detection of specific motif of interruptions. Primer sequences are shown in Table 4. TP-PCR was performed according to published protocols [18,25]. Amplified products were separated by capillary electrophoresis performed on the ABI3130 Genetic Analyzer (Applied Biosystems by Thermo Fisher Scientific, Waltham, MA, USA) using the GeneScan™ 500 LIZ^®^ (Thermo Fisher Scientific, Waltham, MA, USA) as an internal size standard. Any aberrations in the TP-PCR profiles, analyzed using GeneMapper 6 (Applied Biosystems by Thermo Fisher Scientific, Waltham, MA, USA) (discontinuous peak pattern with remarkable gaps and/or unusual drops in peak intensity) were indicative of the existence of variant repeats.

### 4.3. Sanger Sequencing of LR-PCR and TP-PCR Products

The molecular characterization of the *DMPK* expanded alleles containing variant non-CTG repeats has been carried out using a combination of LR-PCR and TP-PCR. Specifically, alleles with small expansions were amplified by LR-PCR following the previously reported protocol [34]. The PCR product was loaded onto a 2.5% agarose gel and the band respective to the expanded allele was excised and purified with QIAquick Gel Extraction Kit (Qiagen, Hilden, Germany). The interruption motifs present in *DMPK* expanded alleles that could not be amplified by LR-PCR were characterized by sequencing of TP-PCR, making only part of the total sequence of the CTG repeat trait and the interruption motif detectable. PCR products were purified using the ExoSAP protocol. Finally, products were directly sequenced with P1_Rev (3′ TP-PCR) and P2_Fw (5′ TP-PCR) using the Big Dye Terminator Cycle Sequencing Kit v3.1 (Thermo Fisher Scientific, Waltham, MA, USA) and visualized by capillary electrophoresis on the Applied Biosystems 3500 Genetic Analyzer.

### 4.4. Methylation Profile of DMPK Regions Flanking the CTG Repeated Array

**Figure 6:**
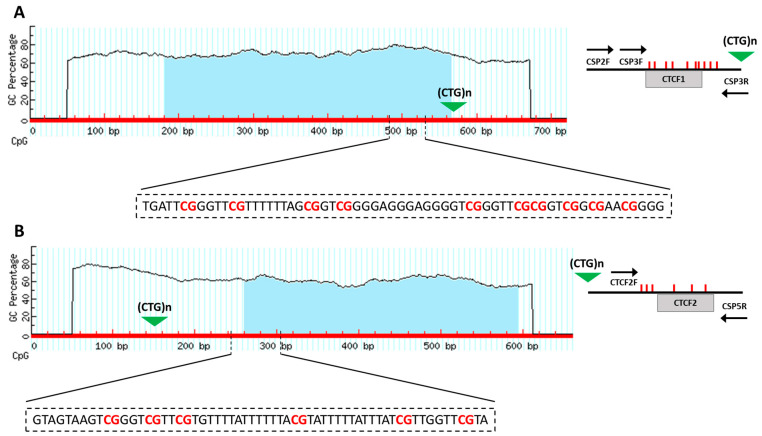
Methylation analysis of CpG islands located in DM1 locus. (**A**) CpG island (blue region) was identified by MethPrimer program at 5′ of the CTG array and location of the CpG sites respect to CTCF binding site 1 (CTCF1) in *DMPK* gene. (**B**) CpG island (blue region) was identified by MethPrimer program at 3′ of the CTG array and location of the CpG sites respect to CTCF binding site 2 (CTCF2) in *DMPK* gene.

Genomic DNA was extracted from 500 µL of anti-coagulated peripheral blood using a Flexigene DNA Kit (Qiagen, Hilden, Germany) and diluted to a final volume of 25 μL with double-distilled water. Subsequently, 1 μg of DNA was treated with bisulphite, which converts all unmethylated cytosines to uracil, leaving the methylated cytosines unaffected, using the EZ DNA Methylation-Gold Kit (Zymo Research, Irvine, CA, USA) according to the manufacturer’s instructions. The methylation study was carried out on two CpG islands identified through the online platform MethPrimer). The first CpG island is located at the 5’ end of the CTG repeats and comprises 10 CpG sites (Figure 6A), whereas the second CpG island is at the 3’ end of the CTG repeats and comprises 6 CpG sites (Figure 6B). A 180 bp fragment of 5′ end region was amplified by PCR using CSP-2F (5-GGAAGATTGAGTGTTCGGGGTA-3) and CSP-3R (5′ biotinylated-CATTCCCGACTACAAAAACCCT-3) primers. The 3′ region has been PCR-amplified in a 178 bp fragment obtained with CTCF-2F (5′-TAAATTGTAGGTTTGGGAAG-3′) and CSP-5R (5′ biotinylated-TTTAACAAAAACAAATTTCCC -3) primers. PCR conditions were 95 °C for 15 min, followed by 45 cycles of 94 °C for 30 s, 56 °C for 30 s and 72 °C for 30 s with final extension of 10 min at 72 °C. Biotinylated PCR products were then processed using the PyroMark Q24 Vacuum Workstation (Qiagen, Hilden, Germany), and subsequent pyrosequencing was performed on a PyroMark Q24 pyrosequencer with PyroMark Gold Q24 Reagents (Qiagen, Hilden, Germany) and the following sequencing primers: CSP-3F (5′-GGGTTTTCGTTTAGTTTTAGTTTTG-3′) for 5′ end region, while CTCF-2F for 3′ end region. The methylation percentage of each CpG region was quantified using the PyroMark Q24 software, version 2.0.7 (Qiagen, Hilden, Germany).

### 4.5. Statistical Analyses

Data were analyzed with GraphPad Prism 7.0 (GraphPad Software, Inc., La Jolla, CA, USA). Before statistical procedures were applied, the assumptions of normality were checked for each variable using the D’Agostino-Pearson test. A non-parametric Mann–Whitney U-test was used for variables showing a skewed distribution, whereas data following a normal (Gaussian) distribution were processed with an unpaired Student’s t-test. Differences were considered significant when the *p* value was <0.05 *, *p* < 0.01 **, *p* < 0.001 ***.

## Figures and Tables

**Table 1:** Main characteristics of DM1 patients carrying *DMPK* interrupted alleles.

Code	Gender	Parental Origin of Mutation	Expanded Allele Length	Family Relationship
Pt 1	M	NA	190	NA
Pt 2	F	Paternal	95–150	Mother of Pt 3
Pt 3	F	Maternal	80–140	Daughter of Pt 2
Pt 4	F	Maternal	230	NA
Pt 5	F	Maternal	94	NA
Pt 6	M	Paternal	175	NA
Pt 7	F	Maternal	186–373	NA
Pt 8	M	Maternal	469–990	Brother of Pt 9, Father of Pt 10
Pt 9	M	Maternal	438–900	Brother of Pt 8, Father of Pt 11
Pt 10	F	Paternal	242–355	Daughter of Pt 8
Pt 11	F	Paternal	248	Daughter of Pt 9
Pt 12	F	Paternal	740–930	Mother of Pt 13
Pt 13	F	Maternal	416	Daughter of Pt 12
Pt 14	M	Paternal	595	Father of Pt 15
Pt 15	F	Paternal	259–632	Daughter of Pt 14
Pt 16	F	Paternal	600–700	NA
Pt 17	M	NA	214–255	NA
Pt 18	F	Paternal	145–221	NA
Pt 19	M	NA	270–745	NA
Pt 20	M	Paternal	400–580	NA

Expanded allele length was determined using LR-PCR followed by Southern blotting analysis of LR-PCR products. Abbreviations: F, Female; M, Male; LR-PCR, Long Range-PCR; NA, Not Available.

**Table 2:** Sequence of *DMPK* expanded alleles containing VRs at 3′ and 5′end of the CTG array. Sequences of TP-PCR products are only partial whereas those obtained by sequencing of LR-PCR products represent the entire expanded allele amplifiable with LR-PCR.

Code	Interrupted End	Sequence	Source
Pt 1	5’	CTG[63]**CCG**[51]CTG[221]	LR-PCR
Pt 2	5’	CTG[6]**CTC**[3]CTG[38]	TP-PCR
Pt 3	5’	CTG[17]**CTC**[3]CTG[152]	LR-PCR
Pt 4	5’	CTG[30]**CCG**[2]CTG[2]**CCG**[1]CTG[105]	LR-PCR
Pt 5	5’	CTG[17]**CGG**[1]CTG[5]**CCG**[2]CTG[2]**CCG**[1]CTG[90]	LR-PCR
Pt 6	5’	CTG[8]**CCG**[1]CTG[5]**CCG**[2]CTG[1]**CCG**[4]CTG[2]**CCG**[4]CTG[1]**CCG**[2]CTG[2]**CCG**[1]CTG[9]	TP-PCR
Pt 7	5’	CTG[14]**CCG**[1]CTG[4]**CCG**[1]CTG[2]**CCG**[1]CTG[248]	LR-PCR
Pt 8	3’	CTG[250]**CCG**[1]CTG[2]**CCGCTG**[2]CTG[5]**CCG**[2]CTG[6]	TP-PCR
Pt 9	3’	CTG[83]**CTC**[4]CTG[60]**CTC**[1]CTG[31]**CCG**[1]CTG[7]**CCG**[1]**CTC**[5]CTG[2]	TP-PCR
Pt 10	3’	CTG[173]**CCG**[1]CTG[2]**CCG**[1]CTG[2]**CCG**[1]CTG[1]**CCG**[1]CTG[2]**CCG**[1]CTG[4]**CCG**[1]CTG[7]**CCG**[1]CTG[7]**CCG**[1]CTG[7]**CCG**[1]CTG[13]	LR-PCR
Pt 11	3’	CTG[191]**CCGCTGCTG**[2]**CCG**[1]CTG[4]**CCGCTGCTG**[2]**CCG**[1]CTG[1]**CCG**[1]CTG[2]**CCG**[1]CTG[1]**CCGCTGCTG**[2]**CCG**[1]CTG[4]**CCG**[1]CTG[2]**CCG**[1]CTG[13]	LR-PCR
Pt 12	3’	CTG[82]**CCGCTG**[2]CTG[5]**CCGCTG**[2]CTG[1]**CCGCTG**[4]**CCG**[2]CTG[4]	TP-PCR
Pt 13	3’	CTG[16]**CCG**[1]CTG[2]**CCGCTG**[4]CTG[1]**CCGCTG**[4]CCG[1]**CCGCTG**[4]**CCG**[1]**CCGCTG**[5]CTG[22]	TP-PCR
Pt 14	3’	CTG[2]**CCG**[1]CTG[112]**TTG**[1]CTG[4]	TP-PCR
Pt 15	3’	CTG[158]**CCG**[3]	TP-PCR
Pt 16	3’	CTG[68]**CCG**[9]CTG[9]	TP-PCR
Pt 17	3’	CTG[108]**CCGCTG**[100]CTG[8]	LR-PCR
Pt 18	3’	CTG[221]**CCG**[1]CTG[5]**CCG**[2]CTG[1]**CCG**[2]CTG[1]**CCG**[2]CTG[2]**CCG**[1]CTG[1]**CCG**[2]CTG[1]**CCG**[2]CTG[5]**CCG**[1]CTG[2]**CCG**[1]CTG[5]**CCG**[1]CTG[4]	LR-PCR
Pt 19	3’	CTG[238]**CTC**[1]**CCG**[1]**CTC**[1]CTG[1]**CCG**[1]CTG[3]**CTC**[1]**CTGCTC**[2]CTG[3]**CTC**[1]CTG[1]**CTC**[3]CTG[1]**CCG**[2]CTG[4]**CCG**[5]CTG[6]**CCG**[1]CTG[13]	LR-PCR
Pt 20	3’	CTG[8]**CCGCTG**[17]CTG[2]**CCG**[1]CTG[25]	TP-PCR

Abbreviations: LR-PCR, Long Range-PCR; TP-PCR, Triplet repeat Primed PCR; VR, Variant Repeat.

**Table 3:** Average CpG sites and global methylation levels (%) both upstream and downstream of the (CTG)_n_ expansion in DM1 patients with VRs at 5′ and 3′ ends.

	5′ End CpG Island		3′ End CpG Island		
ID	Mean CpG Sites Meth%	Mean Global Meth%	Mean CpG Sites Meth%	Mean Global Meth%	Parental Origin of Mutation
Pt 1	11.8	5.6 ± 2.8	9.3	7.9 ± 1.3	NA
Pt 2	4.1		9.5		Paternal
Pt 3	5.3		7.2		Maternal
Pt 4	3.7		8.2		Maternal
Pt 5	4.5		7.7		Maternal
Pt 6	5.2		5.7		Paternal
Pt 7	4.7		7.5		Maternal
Pt 8	4.1	5.2 ± 1.5	26.7	13.9 ± 11.2	Maternal
Pt 9	5.9		42.5		Maternal
Pt 10	6.1		17.0		Paternal
Pt 11	4.5		8.0		Paternal
Pt 12	7.3		11.7		Paternal
Pt 13	5.3		25.2		Maternal
Pt 14	7.9		9.2		Paternal
Pt 15	6.9		8.0		Paternal
Pt 16	3.4		7.3		Paternal
Pt 17	5.6		4.8		NA
Pt 18	4.2		6.8		Paternal
Pt 19	2.9		5.5		NA
Pt 20	4.1		7.7		Paternal

Abbreviations: Meth, Methylation; VR, Variant Repeat.

**Table 4:** Sequences of primers used for TP-PCR. Lowercase letters indicate the tail that is not complementary to the repeat sequence.

	Primer Name	Sequence (5’—> 3’)	Recognized Motif
*DMPK*	P1_Rev	FAM-AGCCTGGCCGAAAGAAAGAAAT	-
	P2_Fw	FAM-GAACGGGGCTCGAAGGGTCCTTGTAGCCG	-
	P3	TACGCATCCCAGTTTGAGACG	-
	P4 CAG_5’	tacgcatcccagtttgagacgCAGCAGCAGCAGCAGCA	(CTG)5
	P4 CTG_3’	tacgcatcccagtttgagacgTGCTGCTGCTGCTGCT	(CTG)5
	P4 esa_5’	tacgcatcccagtttgagacgCAGCGGCAGCGG	(CTGCCG)2
	P4 (CGG)_3’	tacgcatcccagtttgagacgCGGCGGCGGCGGCGG	(CGG)5
	P4 (CTC)_5’	tacgcatcccagtttgagacgCAGCAGCAGCAGGAG	(CTC)
	P4 esa_3’	tacgcatcccagtttgagacgCCGCTGCCGCTGCCGCTGCCGCTGCCGCTG	(CTGCCG)5

Abbreviations: TP-PCR, Triplet repeat Primed PCR; Fw, Forward; Rev, Reverse.

## Data Availability

The authors confirm that the data supporting the findings of this study are available within the article and its Supplementary Materials.

## Supplementary Materials

The following supporting information can be downloaded at: https://www.mdpi.com/article/10.3390/ijms241210129/s1.
