# Supplementary material for: In Cis Effect of DMPK Expanded Alleles in Myotonic Dystrophy Type 1 Patients Carrying Variant Repeats at 5′ and 3′ Ends of the CTG Array
Source: Int J Mol Sci. 2023 Jun 14;24(12):10129. doi: 10.3390/ijms241210129 (PMC10299395; doi:10.3390/ijms241210129)
Supplement: Supplementary file 1 [file ijms-24-10129-s001.zip › ijms-2377789-supplementary.pdf]

Table S1. CpG sites and global methylation (%) both upstream (5' end CpG island) and downstream (3' end CpG island) of the $(CTG)_n$ expansion in "pure" adult DM1 patients (Pt 21 – Pt 37; n = 17) and congenital DM1 patients (Pt 38 - Pt 40; n = 3).

	5' end CpG island		3' end CpG island		
**ID**	**Mean CpG sites Meth%**	**Mean Global Meth%**	**Mean CpG sites Meth%**	**Mean Global Meth%**	**Parental origin of mutation**
Pt 21	4.5		6.8		NA
Pt 22	3.3		7.5		NA
Pt 23	3.5		18.7		Paternal
Pt 24	4.1		10.7		Maternal
Pt 25	3.1		15.5		Paternal
Pt 26	3.1		7.5		NA
Pt 27	3.6		7.0		Paternal
Pt 28	3.2		14.5		Maternal
Pt 29	4.0	3.8 ± 1.0	8.2	13.1 ± 4.9	Paternal
Pt 30	3.6		16.7		Maternal
Pt 31	3.3		18.0		NA
Pt 32	3.7		17.0		Paternal
Pt 33	7.6		17.2		Maternal
Pt 34	3.7		11.2		NA
Pt 35	3.2		14.2		Maternal
Pt 36	3.9		22.8		NA
Pt 37	3.9		9.5		NA
Pt 38	45.5		32.2		Maternal
Pt 39	41.9	40.4	21.5	24.6	Maternal
Pt 40	33.9		20.0		NA

Abbreviations: Meth, Methylation; NA, Not Available.
